# Monitoring HIV-1 Resistance across the Globe

**DOI:** 10.1371/journal.pmed.0020117

**Published:** 2005-04-26

As the HIV-1 epidemic continues to grow, mutations in the virus that confer drug resistance are becoming increasingly important in the clinical management of patients worldwide. Of all the different virus subtypes (A, B, C, D, F, G, H, J, and K) and a rapidly increasing number of established and emerging recombinant viruses, it is subtype B that predominates in Western Europe, the United States, and the rest of the industrialized world. Antiretroviral drugs were developed by studying subtype B, and most data on the genetic mechanisms of HIV drug resistance are also from subtype B. However, worldwide, subtype B is in the minority (~10% of the infected population). In Africa, for example, where there is broad viral diversity, there is a greater spread of subtypes, with subtype C being the most common, representing over half of all infections. Although it seems that current drugs—developed against subtype B virus—are active against non-subtype-B virus, one critical issue is whether viruses from some subtypes or particular regions are more likely than others to develop resistance against certain drugs. Another crucial issue is to identify the mutations that confer drug resistance in non-B subtypes. Answering these questions might determine whether initial treatment strategies should be different for people with non-subtype-B viruses, and also could help decide how patients with non-subtype-B virus who fail antiretroviral therapy should be managed.[Fig pmed-0020117-g001]

**Figure pmed-0020117-g001:**
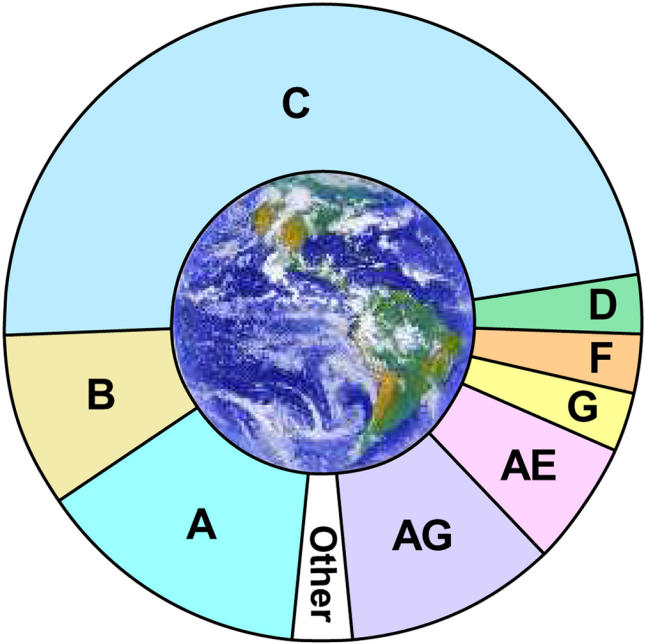
There are many subtypes of HIV-1 worldwide

In a paper in this month's *PLoS Medicine*, Rami Kantor and colleagues from a worldwide collaboration have looked at the mutations found in 3,686 people with non-subtype-B HIV-1 infections compared with those in 4,769 people with subtype B infections. They wanted to answer two questions: first, whether the mutations that cause drug resistance in subtype B viruses also develop in non-subtype-B viruses exposed to antiretroviral drugs, and second, whether novel mutations (i.e., not previously seen in subtype B virus) develop in non-subtype-B viruses when they fail to respond to antiretroviral drugs. What they found was that all of the 55 drug-resistance mutations that have been known to occur in subtype B also occurred in at least one non-subtype-B isolate, and most of these mutations were also statistically associated with antiretroviral treatment in at least one non-B subtype. Conversely, of the 67 mutations associated with antiretroviral therapy in at least one non-B subtype, 61 were also associated with antiretroviral therapy in subtype B isolates.

So it appears that few novel mutations are arising in non-subtype-B viruses exposed to the current antiretroviral drugs and that the present focus on subtype B mutations for global surveillance and genotypic assessments of drug resistance is a reasonable approach. However, the authors emphasize that differences in the types and patterns of drug-resistance mutations are likely to differ between the subtypes, and that larger numbers of samples and further analyses are needed to exclude the possibility of new and/or rare subtype-specific mutations.

